# The Expressions and Mechanisms of Sarcomeric Proteins in Cancers

**DOI:** 10.1155/2020/8885286

**Published:** 2020-06-30

**Authors:** Xiaojing Yang, Hanru Ren, Xiaomao Guo, Chaosu Hu, Jie Fu

**Affiliations:** ^1^Department of Radiation Oncology, Shanghai Jiao Tong University Affiliated Sixth People's Hospital, No. 600, Yishan Road, Shanghai 200233, China; ^2^Department of Orthopedics, Shanghai Pudong Hospital, Fudan University, Pudong Medical Center, Shanghai 201300, China; ^3^Department of Radiation Oncology, Fudan University Shanghai Cancer Center, Shanghai 200032, China; ^4^Shanghai Medical College, Fudan University, Shanghai, China; ^5^Department of Oncology, Shanghai Medical College, Fudan University, Shanghai 200032, China

## Abstract

The sarcomeric proteins control the movement of cells in diverse species, whereas the deregulation can induce tumours in model organisms and occurs in human carcinomas. Sarcomeric proteins are recognized as oncogene and related to tumor cell metastasis. Recent insights into their expressions and functions have led to new cancer therapeutic opportunities. In this review, we appraise the evidence for the sarcomeric proteins as cancer genes and discuss cancer-relevant biological functions, potential mechanisms by which sarcomeric proteins activity is altered in cancer.

## 1. Introduction

The sarcomere represents the basic contractile unit of the myocardium and skeletal muscle. It is composed mainly of actin and myosin. Elastic titin filaments pass through half of each sarcomere, providing unique structural and functional properties to the striated muscle [1]. In addition, accessory proteins form part of the sarcomeric and extrasarcomeric cytoskeleton, such as *α*-actinin (ACTN), myosin-binding protein C (MyBP-C), myomesin, nebulin, desmin, obscurin, and vimentin. These proteins are important to the orderly assembly of actin and myosin filaments into sarcomas and many signaling and transport functions [2]. The cells lose adhesion, integrity, and other morphological characteristics. They acquire the invasion and migration characteristics that lead to cell transformation, which are the most critical steps to initiate cancer metastasis [3]. The expression of many molecules regulating the contraction of actin in metastatic cancer is elevated [4]. Sarcomeric proteins have a predictive value in different types of tumors. Given their pivotal roles in cell growth, metastasis, and tumorigenesis, it appears timely to review what is currently understood about sarcomeric proteins in cancer by addressing the following key questions. What are the molecular functions of their proteins? How do sarcomeric proteins contribute to tumorigenesis? Could their target genes be targeted for cancer therapy? This review summarized the expression and possible mechanisms of sarcomeric proteins in tumors.

## 2. Actin

Actin has basic features in both the nucleus and the cytoplasm. It is involved in many physiological processes, including cell movement, signal transduction, maintaining cell shape, cell adhesion, transcription, and muscle contraction [5]. The expression of actin in the nucleus is related to the cell transcriptional activity. Cell cycle progression and DNA repair require controlled nuclear actin polymerization. Nuclear actin is abnormally expressed in tumors, indicating the importance of this process for cell homeostasis [6]. Six actin isoforms are found in vertebrates. These include *β* and *γ* nonmuscles, *α* bones, *α* hearts, and *α* and *γ* smooth muscle isoactin [7]. Dugina et al. suggested that *β*-actin played a tumor-suppressive role by inhibiting the growth and invasion of cancer cells, while *γ*-actin promoted the development of cancer [8]. *β*-Actin interacts with estrogen receptor *α* (ER *α*) in breast cancer cells after activation and nuclear localization. This interaction may be functionally linked to the expression of ER target genes [9]. Studies have shown a link between cell aging and nuclear actin levels [10]. Mammary epithelial cells maintain senescence by signaling related to laminin-111. The nuclear actin levels increase, and the cells proliferate after interference with the pathway [10]. Laminin-111 regulates exportin 6 through phosphoinositide 3-kinase (PI3K) signal transduction to promote the cytoplasmic localization of actin [11]. Malignant cells are not sensitive to laminin-111 and show high levels of nuclear actin [11]. These high levels help form an estrogen-like phenotype. Since interfering with the signals induced by laminin-111 ultimately leads to transcriptional reprogramming, it is easy to assume that the development of tumors is triggered by nuclear actin-regulated chromatin remodeling. Other potential mechanisms of actin need to be explored.

Cellular actin exists in two forms: monomeric globules called globular-actin (G-actin) and polymer filaments called fibrous actin (F-actin). Under physiological conditions, G-actin is converted into F-actin by ATP. Both are related to the invasion and metastasis of different types of cancers. F-actin protein is highly expressed in breast cancer and related to breast cancer cell metastasis [12]. The inhibition of F-actin can inhibit the aggressiveness of bladder cancer [13]. F-actin expression affects apoptosis, proliferation, and migration of hepatocellular carcinoma cells [14]. In glioma C6 cells, the cellular processes can be regulated by regulating the expression of F-actin and affecting the dynamics of the actin cytoskeleton [15]. These actin changes provide a unique opportunity for new therapeutic strategies. Many studies have focused on targeting actin for cancer therapeutics. Strategies have emerged to inhibit actin expression and to interfere its structure.

## 3. Myosin

Myosin is divided into 12 categories. The largest myosin category is category 2, containing 13 genes, of which 9 genes encode skeletal muscle and cardiac myosin, 1 gene encodes smooth muscle myosin, and 3 genes encode nonmyosin (NM) heavy chain. Category 1 is the second largest category and contains eight independent genes (1A–H). The remaining genes are divided into 10 categories (3, 5, 6, 7, 9, 10, 15, 16, 18, and 19) [16]. Four of the eight genes (MYO1B, MYO1C, MYO1D, and MYO1E) encoding class 1 myosin are widely expressed. MYO1B is of particular concern because it is closely related to metastasis. MYO1B is expressed at high levels in prostate cancer tissues [17], head and neck squamous cell carcinoma (HNSCC) [18], and melanoma [19]. High expression levels of MYO1B may affect the organization of the actin cytoskeleton. In MYO1B-overexpressing cells, the ability of MYO1B to bind to lipids in the plasma membrane and F-actin cytoskeleton may increase cortical actin levels. This may increase the cortical stiffness of these cells, allowing them to migrate more effectively through the extracellular matrix (ECM) *in vivo*. Knocking down MYO1B expression in PC-3 (prostate cancer-3) cells can increase their diffusion area and induce the formation of long and sparse stress fibers, which is speculated to be consistent with the partial reorganization of cortical actin into stress fibers [16]. Two other type 1 myosin isoforms are related to metastasis. MYO1F is highly expressed in cells of the immune system [20]. In infant acute leukemia, exon 7 or exon 9 of the mixed-lineage leukemia (MLL/KMTA) gene is fused to the second exon of MYO1F. The first half of the MLL protein is placed at the N-terminus of MYO1F, which disrupts MLL function and may lead to increased expression of MYO1F in leukemia cells [21]. The loss of MYO1F in neutrophils reduces the levels of cortical actin and increases cell adhesion. Therefore, MYO1F overexpression may have the opposite effect and contribute to the metastatic phenotype in a similar manner as MYO1B. In contrast, MYO1A expression levels usually decrease rather than increase in colorectal tumors [22]. MYO1A is highly enriched in the intestinal microvilli epithelial cells and connects the actin bundles to the plasma membrane, which is very important for the polarization and differentiation of these cells. The loss of MYO1A contributes to the reorganization of the actin cytoskeleton and the dedifferentiation of intestinal epithelial cells, leading to tumor progression [23]. Although MYO1A may help maintain epithelial cells and play a role in suppressing cancer, other type I myosins that promote cell movement, such as MYO1E, may be related to tumor cell dedifferentiation and metastasis [16]. The expression of MYO1A and MYO1E in intestinal epithelium is related to the differentiation status of epithelial cells. The mature cells in crypts have high levels of MYO1E expression, while the mature cells in villi express mainly MYO1A [24]. In addition, MYO1B and MYO1C regulate the post Golgi protein transport or transmembrane protein exocytosis and may help regulate the expression of cell surface growth factor receptors [25, 26]. MYO1C is also involved in regulating the adhesion between epithelial cells, which is an important characteristic of normal epithelial cells [27].

Type 2 myosin subtypes are IIA, IIB, and IIC. During cell migration, myosin II may regulate the traction force exerted by the cell on the substrate, by limiting the formation of liposomes. Cell deformation is essential for cells to migrate through a dense three-dimensional matrix and depends on the activity of type 2 myosin. The inhibition of myosin II can interfere with the passage of glioma cells through the matrix [28]. Type II myosin is related to the regulation of cell–cell adhesion. The loss of intercellular adhesion is usually important in epithelial–mesenchymal transition (EMT). Hence, the depletion of myosin II in epithelial cells may lead to cell dispersion and metastasis. The effects of myosin II on migration, invasion, and metastasis are complex and depend on different isoforms, environments, and cell types. The ablation of myosin IIA in embryonic stem cells leads to a significant reduction in contractility and faster cell migration [29]. Myosin IIA is negatively related to cell proliferation. It inhibits cell migration. The deletion of myosin IIA may lead to cancer metastasis [23]. A high expression level of myosin IIA is related to gastric cancer [30], esophageal cancer [31], and bladder cancer [32], while a decreased expression level is found in squamous cell carcinoma [33]. No change is observed in prostate cancer tissues with low and high metastatic potential [17]. Myosin IIA has been determined as a possible tumor suppressor for squamous cell carcinoma [33]. Myosin IIA not only regulates cell migration but also affects tumor progression by regulating p53 stability and nuclear accumulation [33]. The level of myosin IIA filaments was low in highly metastatic prostate cancer cell line PC-3, indicating that despite myosin IIA expression, the filament formation was inhibited. A reduction in the number of myosin IIA filaments may lead to a reduction in the number of stress fibers and focal adhesions in PC-3 cells. The proportion of total myosin IIA organized into filaments may be important, not the expression level itself. Although the increase and decrease in the myosin IIA level affect the number of myosin IIA filaments and thus the organization of F-actin, other series of proteins can also regulate the degree of formation of myosin IIA in filaments. For example, the transfer-inducing protein S100A4 binds to myosin IIA and dissociates myosin IIA filaments, thereby reducing cell adhesion and increasing cell migration [34]. Myosin IIB is important for cell migration and may help in tumor infiltration. Myosin IIB may promote cancer cell invasion by maintaining cell contractility [23]. Overexpression of myosin IIB was observed in metastatic breast cancer cell lines. However, myosin IIC was expressed in less aggressive breast cancer cell lines [35]. Low expression of myosin IIC in EMT suggested that it was not significant for migration. Myosin IIC may be a negative regulator of cell metastasis [23].

The dimer type 5 myosin isoform has multiple functions. MYO5A and MYO5B are associated with a variety of cancers. The expression level of MYO5A is increased in colorectal cancer, and its interaction with the proapoptotic protein Bmf helps prevent apoptosis, because of a decrease in cell adhesion and invasion of surrounding tissues [23]. Decreased MYO5B levels are associated with gastric cancer [36]. The deletion of MYO5B may affect the integrity of the intestinal brush margin, leading to dedifferentiation and tumor progression in patients with gastric cancer. MYO5C was observed in prostate cancer cell lines [17].

MYO6 differs from other myosins in that it can move toward the tip of actin filaments, rather than toward the barbed end. MYO6 is necessary for collective cell migration related to tumor progression and spread of certain cancers [37]. MYO6 is overexpressed in lung cancer [38], breast cancer [39], hepatocellular carcinoma [40], ovarian cancer [41], and prostate cancer [42]. In prostate cancer cells, MYO6 is mainly associated with recovered endosomes, not endocytic vesicles or Golgi apparatus, which may be due to lower levels of Dab2 and optineurin in these cells. It increases PSA and VEGF secretion, thus increasing cell movement and thereby promoting metastasis [43]. MYO6 expression is directly related to cell aggressiveness. Knocking out MYO6 in prostate cancer cells leads to in vitro migration and defects in growth [42]. MYO6 may regulate the metastasis of cancer cells through its effect on protein transport to the cell surface, hence affecting cell migration. Moreover, MYO6 plays a role in adherent connections of epithelial cells [44]. Therefore, the effect of MYO6 on collective migration might be related to its role in cell-to-cell adhesion.

Type 7 contains two genes, MYO7A and MYO7B. A link between MYO7A and the risk of malignant melanoma has been observed [45]. The role of MYO7A in pigmentation to regulate cell adhesion remains to be determined.

Type 9 contains two genes: MYO9A and MYO9B. MYO9A is highly expressed in various organs [46]. MYO9B is overexpressed in metastatic prostate cancer cells, prostate cancer tumors [17], lung cancer [47], and esophageal cancer [48]. High levels of MYO9B promote actin reorganization by reducing filaments and promote metastasis by promoting the breakdown of stress fibers and reducing cell adhesion to promote the cancer phenotype [17].

The overexpression of MYO10 results in a large number of filamentous pseudopods, which is important for cell migration. The expression level of MYO10 increases in breast cancer [49], lung adenocarcinoma [50], non-small-cell lung cancer (NSCLC) [51], metastatic prostate cancer cell lines, and prostate cancer tissues [17]. This high expression level increases the number of filamentous pseudopods, thus allowing cells to migrate better through the matrix [49]. MYO10 plays a key role in cell phagocytosis and filamentous pseudopod formation [52]. The increase in the number of filamentous pseudopods is associated with an increase in cancer progression, while the upregulation of genes regulating filamentous foot formation is associated with aggressive subtypes of breast cancer [53]. MYO10 is necessary for the growth, migration, and invasion of breast cancer cells in vivo and in vitro [53]. MYO10 cannot support breast cancer cell migration when it lacks the integrin-binding domain [49]. MYO10 interacts with the calmodulin-like protein, which functions as a light chain of MYO10 and is downregulated in tumors [54]. The knockout of MYO10 in PC-3 cells eliminated filamentous pseudopods, reduced cell motility, increased cell spreading area, and triggered the formation of F-actin bundles in the cell center [17].

Class 18 contains two genes, MYO18A and MYO18B. MYO18A is widely expressed, while MYO18B is highly expressed in the striated muscle but is expressed at low levels in other tissues [55]. MYO18A is overexpressed in metastatic prostate cancer cells, which may help reduce NM2A stress fibers [17]. The knockout of MYO18A in PC-3 cells increased the area of cell proliferation and significantly increased the formation of NM2A filaments in plate-like concentric tissues, which was consistent with its effect on NM2 filament formation. However, MYO18A is not overexpressed in prostate cancer tissues and has not been reported in other cancers. MYO18B is a tumor suppressor gene whose deletion or mutation is associated with malignant pleural mesothelioma [56], ovarian cancer [57], lung cancer [58], melanoma, and pancreatic cancer [59]. The recovery of MYO18B expression in lung cancer cell line H1299 inhibited cell growth and movement through interaction with HOMER2 [60]. While it has been documented with inhibitors effective in vitro, evidence for in vivo effectiveness is still lacking.

## 4. Titin

Elastic TTN pass through half of each sarcomere, providing unique structural and functional properties to the striated muscle [1]. TTN is frequently mutated in many types of tumors, including NSCLC and colon adenocarcinoma [61]. Studies have shown that TTN mutations have predictive significance for solid tumors. The clinical significance of TTN mutations has been shown in many cohort studies on cancers treated with immune checkpoint blockade, including NSCLC, melanoma, and other solid tumors [62]. TTN mutations in breast cancer have not shown a strong discriminatory effect on patient survival. Patients with mutations in certain cancer-related genes have a good prognosis, and these mutations are completely mutually exclusive with TTN/TP53 conversion [63]. TTN and TP53 double mutations may be involved in breast cancer research through the downstream pathway and signaling network. TTN deletion mutation has a positive prognosis for lung squamous cell carcinoma but has nothing to do with lung adenocarcinoma. TTN/TP53 double mutation also has a good prognosis and predictive value for lung squamous cell carcinoma [64]. With the popularity and development of sequencing technologies, further clinical trials are needed to explain their relevance and significance.

## 5. MyBP-C

There are three paralogs in MyBP-C: MyBPC-1, MyBPC-2, and MyBPC-3 [65]. MyBPC-1 is highly expressed in laryngeal squamous cell carcinoma [66]. An increased expression level of the MyBPC1 gene was observed in breast cancer [67]. This gene is speculated to be involved in progesterone signaling related to the progression of hormone-related cancers [68]. The expression level of MyBP-C-1 also increased in prostate cancer [69]. MyBPC-2 and MyBPC-3 have not been reported in tumors.

## 6. Obscurin

**Figure 1:**
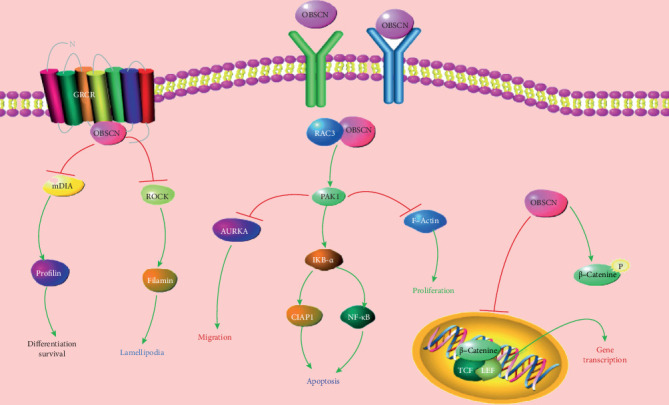
The relevant pathways for cancer caused by *OBSCN.*

Obscurin is encoded by a single *OBSCN* gene. Balakrishnan et al. evaluated gene mutations in glioblastoma, melanoma, and pancreatic ductal adenocarcinoma. They found that the *OBSCN* gene was mutated in melanoma and glioblastoma [70]. Price et al. reported a single gene set consisting of OBSCN and C9orf65 that distinguished gastrointestinal stromal tumors and leiomyosarcoma tumors. These studies demonstrated a close relationship between the *OBSCN* gene and cancer. After genetic analysis of breast and colorectal cancers, *OBSCN* was found to be one of the most common mutant genes. Perry et al. determined the role of obscurins in cancer development by regulating cell survival [71]. They analyzed obscurins in breast, colon, and skin cancer cell lines. The Western blot analysis displayed that the expression level of obscurin protein is significantly reduced in cancer cells due to reduced mRNA levels and mutant transcripts. Nontumorigenic MCF10A breast epithelial cells showed higher viability and reduced apoptosis after transduction with obscurin shRNAs. Subsequently, antiapoptotic genes BAG4 and HAX1 were upregulated, while caspase-9 and caspase-3 were downregulated. The downregulation of obscurins reduces the sensitivity of normal breast epithelial cells to apoptosis, providing them with a survival advantage. Obscurins may help suppress tumors. Decreased expression levels of *OBSCN* largely interfere with cell integration and activate cancer. Hence, *OBSCN* may act as an oncogene and prevent cell migration [72]. Some studies on *OBSCN* gene mutations have shown that obscurin has a potential role in glioblastoma, melanoma, pancreatic cancer [70], colorectal cancer, and breast cancer [73]. *OBSCN* gene mutations have affected various interconnected proteins related to cell adhesion and integration processes. *OBSCN* mutations have a significant effect on the expression of E-cadherin, *β*-catenin, and *α*-catenin. This expression initiates the dispersion of epithelial cells and then increases F-actin levels, subsequently causing cell migration [74]. *OBSCN* mutations are responsible for the decline in the expression of important proteins [71]. Protein kinases are related to various cellular functional mechanisms. Serine/threonine kinase (STK) is an important protein kinase, which is related to cell proliferation, differentiation, and apoptosis. STK regulates cadherin-promoted intercellular adhesion by binding to p120-catenin [75]. *OBSCN* mutations can severely affect STK protein activity and also act as a ligand for small ankyrin (sANK1). sANK1 is an essential protein of the essential spectrin–actin backbone, which is involved in the activation and proliferation of cell movement and causes metastasis [76]. A large amount of evidence suggests that *OBSCN* mutations may be closely related to Wnt signaling regulated by *β*-catenin and other activators [77]. In the nucleus, *β*-catenin binds to transcriptional activation elements, thus regulating cell proliferation and differentiation [78]. *OBSCN* mutations mainly affect the downstream signals of *β*-catenin, stimulating many types of cancers [79]. *OBSCN* is considered an indirect regulator of fibronectin. Fibronectin can regulate signal transduction, cell proliferation, and cytoskeleton reorganization [80]. In metastatic cancer, mutations in *OBSCN* and Ras protein were observed. These proteins, including RHOD, RHOT2, and RAC3, are involved in the reorganization of the actin cytoskeleton and GTPase activity [81]. RAC3 is an important gene for activating PAK1; it is essential for stimulating F-actin formation and causing cell migration. PAK1 activates another important proliferation-related protein and inhibits apoptosis through NF-*κ*B [82]. *OBSCN* is involved in cell regulatory mechanisms, such as cell adhesion, to regulate tumorigenesis. The relevant pathways for cancer caused by *OBSCN* are shown in Figure 1.

## 7. ACTN

ACTN has four subtypes: ACTN1, ACTN2, ACTN3, and ACTN4. ACTN4 is located in the cytoplasm and is involved in cytoskeletal tissue. ACTN4 can translocate into the nucleus and plays multiple roles in signal transduction and gene regulation [83]. ACTN4 interacts with nuclear receptors, such as estrogen receptor alpha (ER*α*), and induces the transcription of its target genes [84]. It can also act as a transcriptional coactivator of nuclear factor *κ*B (NF-*κ*B) and increase the expression of target genes [85]. ACTN4 plays a key role in cell movement and is involved in various cancers. The metastatic ability of ACTN4 in colorectal cancer [86], breast cancer [87], ovarian cancer [88], pancreatic cancer [89], salivary gland cancer [90], and prostate cancer [91] is related to the malignant phenotype. In breast cancer, the knockdown of ACTN4 can reduce the expression of ERa target genes and cell proliferation [87]. *ACTN4* gene amplification is related to poor prognosis and tumor chemoresistance in patients with ovarian cancer [88]. It induces the EMT of colorectal cancer cells by activating the phosphoinositide 3-kinase (PI3K)/Akt signaling pathway [86]. ACTN4 is overexpressed in prostate cancer cell line DU145. The knockdown of ACTN4 obviously reduced the proliferation and invasion of DU145 cells. ACTN4 may serve as a potential therapeutic target for prostate cancer [91]. Patients with high expression of ACTN4 in pancreatic cancer have a worse prognosis after treatment with chemotherapy and radiation [92]. The amplification of ACTN4 was inversely related to the survival of patients with small cell lung cancer [93]. The knockdown of ACTN4 in gastric cancer cells obviously increased cell-matrix adhesion and reduced gastric cancer cell migration and invasion. NF-*κ*B was downregulated after the knockdown of ACTN4 in gastric cancer cells. ACTN4 was significantly upregulated in patients with metastatic gastric cancer. ACTN4 can reduce cell adhesion and enhance the migration and invasion of gastric cancer cells, hence serving as a new therapeutic target for gastric cancer [94]. In addition, ACTN4 is involved in cell movement and proliferation of cervical cancer cells. It promotes EMT and cell cycle progression in cervical cancer through *β*-catenin stabilization [95]. Cancer stem cells (CSCs) cause tumors and are self-renewing and differentiating. Chemical resistance, tumorigenesis, and EMT are representative characteristics of CSCs [96]. Jung et al. demonstrated that ACTN4 plays a key role in promoting the chemoresistance of cervical cancer [97].

*ACTN4* and *ACTN1* are genes with a close functional overlap. ACTN4 plays a key role in cell proliferation, while ACTN1 regulates cell movement. Its overexpression causes the loss of cell adhesion and metastasis. Increased expression levels of ACTN4 and ACTN1 played a role in pancreatic intraepithelial neoplasia–pancreatic ductal adenocarcinoma [98]. During the occurrence and development of astrocytoma, ACTN1 and ACTN4 are regulated differently, and their effects on the malignant development of astrocytoma cells are opposite [99]. Kovac et al. determined that the expression level of ACTN1 frequently increases in human breast cancer and is related to a poor prognosis. In breast cancer cells, increased levels of ACTN1 cause unstable adhesion, thus promoting the collective migration of cancer cells [100]. In this regard, novel therapies targeting ACTN may best be applied in the future for clinical therapies.

## 8. Nebulin

Nebulin plays a key role in cell adhesion and actin filament structure [101]. The first member of the nebulin family, LASP1, is a mixed lineage leukemia (MLL) fusion partner [102]. The second member of the nebulin family, LASP2, is related to the construction of the cytoskeleton structure [101]. LASP2 is found to be associated with MLL in hematological disorders in infants. LASP2 overexpression promotes cell migration while inhibiting cell invasion [103]. LASP1 and LASP2 have similar domains and play roles in a variety of cancers, including lung cancer [104], bladder cancer [105], gallbladder cancer [106], prostate cancer [107], cholangiocarcinoma [108], and hepatocellular carcinoma [109]. LASP1 regulates the proliferation of cervical cancer through the PI3K/Akt pathway [110]. Hosseini et al. [111] reported that the expression of LASP2 was upregulated in colorectal cancer tissues. However, Wanget et al. [112] showed that LASP2 attenuated the growth and migration of colorectal cancer. LASP2 promotes the migration and invasion of NSCLC cells [104]. LASP2 is highly expressed in cervical cancer [113]. LASP2 knockdown can inhibit cervical cancer cell proliferation, migration, and invasion. It is suggested that LASP2 knockdown may have an inhibitory effect on tumor metastasis. LASP2 knockdown in cervical cancer cells significantly inhibits the PI3K/Akt pathway. LASP2 upregulation inhibits the migration and invasion of pancreatic cancer cells by inhibiting TGF-*β*-induced EMT [114]. LASP2 inhibits the malignancy of bladder cancer by inhibiting the activation of the Wnt/*β*-catenin signaling pathway [105]. These roles of LASP2 in tumors may be attributed to different races and sample sizes. These findings indicate that LASP2 serves as an oncogene or suppressor in cancers. It is expected, even with promising preclinical responses to targeting LASP2, that tumor types and context will add to the complexity and heterogeneity of response to any one strategy.

## 9. Desmin

Desmin is a blood vessel–related pericyte marker throughout the angiogenesis process. As a result of angiogenic signals, pericytes are recruited into the developing endothelial tube; they form a stable sheath around the surrounding new vessels as they mature and elongate and express desmin in increased amounts. Mature pericytes are embedded in the basement membrane of adjacent endothelial cells, which are essential for normal physiology and angiogenesis in cancer [115]. Desmin is reported to be expressed in osteoblastic melanoma [116]. Desmin-expressing melanoma may be diverse in immunophenotype and ultrastructure. Most cases reported by Smith et al. showed nodular hyperplasia and rhabdoid or smooth muscle transformation [117]. Of the nine proliferative melanomas, seven were positive for desmin. Further, 10 of the other 10 unspecified melanomas were positive for desmin. These desmin-expressing melanomas are located in the head and neck [118]. Desmin is a marker of striated muscle cells. This indicates that tumors also have rhabdomyoblastic differentiation. Another possibility is that desmin is involved in the regulation of DNA transcription and gene expression [118]. Its presence may indicate higher malignancy and worse prognosis. Takeda et al. [119] reported nasopharyngeal anaplastic plasmacytoma with desmin expression. Arentz et al. [120] found that desmin was overexpressed in colorectal tumors relative to normal mucosa. The desmin expression level significantly increased in stage III tumors compared with stage I and II tumors, indicating that the degree of proliferation is higher in advanced tumor tissues.

## 10. Synemin

Synemin has three splice variants (*α*, *β*, and L). Synemin is found in all muscle cell types, a few nerve cell types, and various other nonepithelial cell types. In astrocytoma cells, synemin regulates Akt phosphorylation by interacting with PP2A to regulate proliferation [121]. Synemin is expressed in astrocytoma cells, but not in mature astrocytes. Some glioblastomas express high levels of synemin [122]. In astrocytoma cells, synemin enriches the membrane domains involved in cell movement. Synemin is one of the prognostic factors for patients with ovarian cancer receiving platinum-based chemotherapy drugs [121]. In normal breast epithelial cells, the expression level of synemin increases due to the methylation of the promoter of the synemin gene but drastically decreases in breast cancer. Synemin is a feasible tumor suppressor, and its promoter methylation status can predict the risk of recurrence in patients with breast cancer [123]. Synemin is an intermediate filament–related protein playing a role in the formation of hepatocellular carcinoma, and changes in its expression level may lead to tumor cell polymorphisms [124]. New knowledge indicates that there are new treatment opportunities.

## 11. Plectin

Plectin serves as a bridge between actin filament and intermediate filament networks. Its main role is to act as a structural linker between the cell membrane and cytoskeleton components [125]. Through these interactions, plectin is important in cell–cell interaction, cell–ECM interaction, migration, and cell integrity and plasticity. Fibroblasts lacking lectin showed slower actin recombination and decreased exercise capacity. Plectin-1 knockout showed defects in cell migration into fibroblasts and leukocytes [126]. Studies showed that plectin was related to cancer progression and metastasis. Plectin-1 has been shown as a potential biomarker for invasive papillary mucinous tumors in the pancreatic duct [127]. Raymond et al. [128] showed that plectin was highly expressed in NSCLC cell lines. These plectin-positive subpopulations are highly clonable and enrich tumor cell migration and other characteristics. Lorna et al. [129] reported that the reduced expression level of plectin impaired the invasion and adhesion of colon cancer cells. In prostate cancer cells, the downregulation of plectin inhibited cell proliferation, migration, and invasion. The upregulation of plectin positively correlates with the invasion and metastasis of androgen-independent prostate cancer [130]. The findings of Koji et al. indicated that plectin was significantly overexpressed in HNSCC. Plectin helps in the migration and invasion of HNSCC cells by activating Erk1/2 kinase. It is a potential prognostic biomarker for HNSCC. The survival rates for patients with high expression levels of plectin decreased significantly. The knockdown of plectin expression restrained the migration and invasion of HNSCC cells [131]. The inhibition of plectin impaired the migration of breast cancer MCF-7 cells [132]. Plectin interacts with integrin *β*4, which is a receptor of laminin [133]. Integrin *β*4 is related to cell migration and invasion. The high expression level of integrin *β*4 leads to poor prognosis in a variety of cancers. Therefore, the overexpression of plectin may promote the migration and invasion of HNSCC cells by binding to integrin *β*4. However, Osmanagic-Myers et al. obtained opposite results showing that inhibiting plectin increased the migration of keratinocyte [134]. In keratin, when plectin is depleted, the MAP kinase Erk1/2 can positively regulate the migration of keratinocytes. Ding et al. suggested that the knockdown of lectin attenuated Erk1/2 activation [135]. The data indicated that the effect on the migration of plectin-deficient cells depended on Erk1/2 activation. Plectin is a useful biomarker in clinical treatment and can be used for more effective postoperative treatment options. For example, radiation alone can be used for treating patients with low plectin levels, while a combination of radiation with chemotherapy is used for patients with high plectin levels [131]. The use of liposome chemotherapeutics targeting plectin has improved the treatment of ovarian cancer [136]. Experimental evidence supporting the plectin in animal models suggests that some human cancers could also be related to plectin and hence could be treated by targeting plectin.

## 12. Nesprins

The nesprin family consists of Nesprin-1, Nesprin-2, Nesprin-3, and Nesprin-4, which are encoded by the SYNE1, SYNE2, SYNE3, and SYNE4 genes, respectively. Nesprin-1, Nesprin-2, and Nesprin-3 affect centrosome localization, cell morphology, and migration [137]. *SYNE1* gene mutations cause muscular dystrophy, cerebellar ataxia, arthritis, and cancer. During tumor formation, proliferation, cell movement, metabolism, adhesion, and DNA damage responses are abnormal. These characteristics depend on the organization of the nuclear envelope (NE), whose morphological changes are a hallmark of cancer. Nesprin-1 expression is downregulated in several cancers. *SYNE1* mutations were detected in ovarian and colorectal cancers [73, 138]. The *SYNE1* gene is frequently methylated in lung cancer cell lines [139]. The *SYNE1* gene was found to be one of the genes involved in the progression of glioblastoma [140]. The expression level of Nesprin-1 decreased in liver cells. Nesprin-1 defects are related to changes in the nuclear morphology, nuclear membrane structure, and centrosome localization of liver Hep3B and Huh7 cells [141]. Previous reports pointed out that the misplacement of Nesprin-1 in HeLa and Swiss 3T3 cells resulted in the softening of the cytoplasm and impaired connections between the nucleus and the cytoskeleton, leading to cancer [142]. Nesprin-1 deficiency causes increased cellular senescence. Defective cells in NE show increased aging [143]. Cancer cells can age due to the shortening of telomeres, DNA damage, and oxidative stress. DDR networks in Nesprin-1-deficient cells may serve as a trigger for cell aging. The accumulation of DNA damage can cause premature aging [144]. Nesprin-1 interacts with the cytoskeleton in the cytoplasm, which is significant for the shape and size of the nucleus and for the proper maintenance of the cytoskeleton structure. Overexpression of Nesprin-1 can change the phenotype of tumor cells by enhancing nuclear structure, NE tissue, centrosome localization, and genomic instability. King et al. studied the role of nesprins in endothelial cells. They found that the depletion of Nesprin-1 or Nesprin-2 increased filamentous actin levels but reduced migration and angiogenesis [145]. Angiogenesis helps in the development of cancer. Nesprin-2 can also interact with dynein Lis1 to coordinate with the ECM to regulate metastasis and invasion [146]. Nesprin-2 depletion affects NF-*κ*B and BRCA1 nuclear localization. Nesprin-2 has an impact on the abnormal localization of nuclear proteins in ovarian adenocarcinoma cells [147]. Nesprin-3 binds to intermediate filaments, which is essential for mechanotransduction. Interestingly, the deprivation of Nesprin-3 induces the elongation of aortic endothelial cells and is related to migration and polarization [148]. No studies exist on the role of Nesprin-4 in tumors. It is hoped that nesprins may help in future cancer research and treatment.

## 13. Vimentin

Vimentin is a typical marker of EMT. During EMT, epithelial cells acquire a mesenchymal phenotype. Consequently, they significantly change their shape and show enhanced mobility. During the inverse process of EMT, the cells exhibit an epithelial phenotype, the expression of vimentin decreases, and the rate of cell movement decreases. Studies on stem cells have shown vimentin to be important for tumor growth [149]. *In vitro* studies have shown that vimentin may function as a tumor promoter [150]. Increased expression levels of vimentin have been reported in various cancers, including breast cancer, prostate cancer, endometrial cancer, central nervous system tumors, gastrointestinal tumors, and malignant melanoma [150]. In prostate cancer, vimentin is expressed in poorly differentiated cancers, but hardly detectable in highly differentiated or moderately differentiated tumors [151]. The downregulation of vimentin in prostate PC-3 cells leads to an important reduction of cell motility and invasive activity [151]. Vimentin is overexpressed in the highly invasive prostate cancer cell line CL1. After silencing vimentin expression, the cells displayed a marked decrease in infiltration [152]. Vimentin is highly expressed in the metastatic prostate PC-3M-1E8 cells and regulates the aggressiveness of these cells through E-cadherin/*β*-catenin complex [153]. In gastric cancer, vimentin often plays a role in the invasive phenotype and as a prognostic marker of gastric cancer [154]. Vimentin expression is positively related to lymph node metastasis in esophageal cancer [155]. And the expression of vimentin is related mainly to the metastasis of hepatocellular carcinoma [156]. Vimentin gene methylation often occurs in advanced colorectal cancer [157]. Vimentin methylation can be used as a biomarker for colorectal cancer. The overexpression of vimentin in colorectal cancer is related to stromal components, microvascular-lined endothelial cells, and tumor-infiltrating lymphocytes [158]. Vimentin has been shown to increase pancreatic cancer cell invasion, while silencing vimentin cells reduces invasiveness [159]. The expression level of vimentin increases in highly aggressive breast cancer cell lines [160], and this overexpression is closely related to migration and invasion. Vimentin plays a key role in the EMT of breast cancer, and its knockdown results in a reduction in genes associated with invasion and breast basal-like phenotype [161]. High expression of vimentin in malignant melanoma tumor can be used as a diagnostic and metastasis marker [162]. In glioma, vimentin expression appears to be related to cell density and chemoradiotherapy; it is detected mainly in low-density cell cultures [163]. Specific phosphorylated forms of vimentin can be used to detect the migration potential and distinguish types of meningiomas [164]. The overexpression of vimentin is a predictor of survival among patients with NSCLC [165]. Glycosylated vimentin was found to be downregulated in lung adenocarcinoma; it is considered to be a biomarker for the diagnosis and treatment [166]. Vimentin can serve as a potential diagnostic tool for detecting cancer. In addition, the overexpression of vimentin during transfer suggests that it has a transfer effect.

## 14. Conlusions

Individual sarcomeric proteins are potential regulators of a variety of physiological functions. However, the true function of sarcomeric proteins needs to be further elucidated in addition to maintaining the structural function of cells. In most cancers, sarcomeric proteins are abnormally expressed. The overexpression of multiple sarcomeric proteins has been linked to the aggressiveness of cancer. We have summarized it in Table 1. Importantly, sarcomeric proteins are closely related to metastatic phenotype and poor prognosis. Understanding the mechanism of sarcomeric protein regulation and the theory of sarcomeric proteins on the cell surface may help better understand cancer and provide better ways for controlling the aggressiveness of cancer cells. All findings suggest that sarcomeric proteins may become clinically relevant biomarkers for different cancers. However, more research is needed to assess the main function of sarcomeric proteins in tumorigenesis. Given the available data, sarcomeric proteins are likely to become attractive and promising cancer treatment targets and develop the potential to serve as novel clinical prognosis and diagnostic tools. In addition, the use of sarcomeric protein–specific chemical inhibitors and novel therapeutic drugs must be encouraged.

## Figures and Tables

**Table 1:** Association of sarcomeric proteins expressions with cancers.

Protein	Gene	Cancer (references)	Overall deregulation	Association
Actin				
*β*-Actin		Breast cancer [9]	↑	Estrogen receptor *α*
F-Actin		Breast cancer [12]	↑	Coronin 1c
		Bladder Cancer [13]	↑	Piperlongumine
		Liver cancer [14]	↑	ROCK1
		Glioma [15]	↑	RhoA/ROCK
Myosin				
	MYO1B	Prostate cancer [17]	↑	Organization of the actin cytoskeleton
		Head and neck squamous cell carcinoma [18]	↑	NR
		Melanoma [19]	↑	NR
	MYO1F	Acute myeloid leukemias [21]	↑	Thet (11;19) (q23;p13) fusing MLL
	MYO1A	Colorectal cancer [22]	↓	Actin bundle and plasma membrane
Myosin IIA		Gastric cancer [30]	↑	cJun N-terminal kinase signaling pathway
		Esophageal squamous cancer [31]	↑	Cell migration
		Bladder cancer [32]	↑	NR
		Squamous cell carcinomas [33]	↓	P53
Myosin IIB		Breast cancer [35]	↑	Ribonucleoprotein-E1
Myosin IIC		Breast cancer [35]	↑	Ribonucleoprotein-E1
	MYO5A	Colorectal cancer [23]	↑	Snail, Bmf
	MYO5B	Gastric cancer [36]	↑	Rab11a
	MYO5C	Prostate cancer [17]	↑	Actin organization and cell morphology
	MYO6	Lung cancer [38]		ERK1/2
		Breast cancer [39]		NR
		Hepatocellular carcinoma [40]		PRAS40, p38
		Ovarian cancer [41]	↑	NR
		Prostate cancer [42]	↑	Androgen receptor
	MYO7	Melanoma [45]	↑	NR
	MYO9B	Prostate cancer [17]	↑	Organization of the actin cytoskeleton
		Lung tumor [47]	↑	SLIT/ROBO
		Esophageal cancer [48]	↑	Non muscle myosin 2
	MYO10	Breast cancer [49]	↑	P53
		Lung adenocarcinoma [50]	↑	NR
		Non-small-cell lung cancer [51]	↑	NF-kappaB, miR-124
		Prostate cancer [19]	↑	Organization of the actin cytoskeleton
	MYO18B	Prostate cancer [17]	↑	Organization of the actin cytoskeleton
		Pleural mesothelioma [56]	↓	NR
		Ovarian cancer [57]	↑	NR
		Lung cancer [58]	↑	HOMER2
		Glioblastoma, melanoma, pancreatic carcinoma [59]	↑	NR
Titin		Lung squamous cell carcinoma, lung adenocarcinoma, and colon adenocarcinoma [61]	↑	P53/KRAS
MYBPC1		Breast cancer [67]	↑	Progesterone signaling
		Prostate cancer [69]	↑	NR
Obscurin		Glioblastoma, melanoma, and pancreatic carcinoma [70]	↓	*β*-Catenin
		Breast cancer, colon cancer [74]	↓	*β*-Catenin
*α*-Actinin-4		Colorectal cancer [86]	↑	LIM kinase 1
		Breast cancer [87]	↑	Estrogen receptor *α*
		Ovarian cancer [88]	↑	19q12-q13
		Ductal carcinoma of the pancreas [89]	↑	19q13.1-2
		Salivary gland carcinoma [90]	↑	NR
		Prostate cancer [91]	↑	NR
		Non-small-cell lung cancer [93]	↑	NR
		Gastric cancer [94]	↑	NF-*κ*B
		Cervical cancer [97]	↑	Snail, beta-catenin
		Pancreatic ductal adenocarcinoma [98]	↑	Fascin-1
		Astrocytoma [99]	↑	NR
*α*-Actinin-1		Pancreatic ductal adenocarcinoma [98]	↑	Fascin-1
		Astrocytoma [99]	↓	NR
		Breast cancer [100]	↑	E-cadherin
LASP1		Acute myeloid leukemia [102]	↑	t(11;17)(q23;q21)
LASP2		Non-small-cell lung cancer [104]	↑	Phosphorylation of FAK
		Bladder cancer [105]	↑	Wnt/*β*-catenin signaling pathway
		Gallbladder cancer [106]	↑	G2/M phase
		Prostate cancer [107]	↑	NF-kappaB pathway
		Cholangiocarcinoma [108]	↑	NR
		Hepatocellular carcinoma [109]	↑	Vimentin
		Cervical cancer [110]	↑	PI3K/Akt/mTOR
		Colorectal cancer [111]	↑	Thin filaments
		Colorectal cancer [112]	↓	JNK/p38 MAPK pathway
Desmin		Melanoma [116]	↑	NR
		Colorectal cancer [120]	↑	Vimentin
Synemin		Ovarian cancer [121]	↑	NR
		Astrocytoma [122]	↑	Intermediate filament
		Breast cancer [123]	↑	Zyxin
		Hepatocellular carcinoma [124]	↑	Intermediate filament
Plectin		Pancreatic intraductal papillary mucinous neoplasm [127]	↑	NR
		Lung cancer [128]	↑	NR
		Colon carcinoma [129]	↑	Isoform-specific
		Prostate cancer [130]	↑	Nyalwidhe
		HNSCC [131]	↑	Erk 1/2 kinase
		Breast cancer [132]	↑	Periplakin
		Ovarian cancer [136]	↑	PARP
Nesprin-1		Ovarian cancer [138]	↓	ESR1/SYNE1
		Breast and colorectal cancers [73]	↓	NR
		Lung cancer [139]	↓	Methylation
		Glioblastoma [140]	↓	P53
Nesprin-2		Ovarian cancer [147]	↓	Ca(2+)/calmodulin
Vimentin		Prostate cancer [153]	↑	E-cadherin/*β*-catenin
		Gastric cancer [154]	↑	circNHSL1
		Esophageal squamous cell carcinoma [155]	↑	NR
		Hepatocellular carcinoma [156]	↑	NR
		Colorectal carcinoma [157]	↑	Methylation
		Pancreatic cancer [159]	↑	NR
		Breast cancer [160]	↑	*β*-Catenin
		Melanoma [162]	↑	NR
		Glioma [163]	↑	Cellular density
		Meningiomas [164]	↑	NR
		Lung cancer [165]	↑	NF-kappaB

Abbreviations: NR = not reported.
